# Conotoxins That Could Provide Analgesia through Voltage Gated Sodium Channel Inhibition

**DOI:** 10.3390/toxins7124890

**Published:** 2015-12-10

**Authors:** Nehan R. Munasinghe, MacDonald J. Christie

**Affiliations:** Discipline of Pharmacology, The University of Sydney, Sydney, NSW 2006, Australia; nmun5523@uni.sydney.edu.au

**Keywords:** conotoxins, toxins, Na_V_, ion channels, pain, analgesia, inhibition

## Abstract

Chronic pain creates a large socio-economic burden around the world. It is physically and mentally debilitating, and many sufferers are unresponsive to current therapeutics. Many drugs that provide pain relief have adverse side effects and addiction liabilities. Therefore, a great need has risen for alternative treatment strategies. One rich source of potential analgesic compounds that has emerged over the past few decades are conotoxins. These toxins are extremely diverse and display selective activity at ion channels. Voltage gated sodium (Na_V_) channels are one such group of ion channels that play a significant role in multiple pain pathways. This review will explore the literature around conotoxins that bind Na_V_ channels and determine their analgesic potential.

## 1. Introduction

Chronic pain is a major problem in the world today. The total cost of chronic pain in the United States was estimated to be between $560 and $635 billion per annum [1]. This cost is greater than cancer and heart diseases combined [1]. The current therapeutics that are available for chronic pain often provide only limited pain relief and have many side effects. Therefore, there is a great need for alternative therapies that provide analgesia to chronic pain sufferers. Animal toxins are one such alternative source for pain therapy. In particular, conotoxins from marine cone snails provide a very diverse pool of selective compounds that may form the foundation of future analgesic drugs. Conotoxins interacting selectively with Na_V_ channels have potential as pain therapeutics, as reviewed previously [2,3]. The present review updates and provides the perspectives of the authors on the therapeutic potential of conotoxin Na_V_ channel inhibitors for pain management.

The pain transmission pathway involves sensors known as nociceptors, which transmit the signal from the site of pain to primary sensory neurons in the spinal cord known as dorsal root ganglion (DRG) neurons. Central fibres of nociceptive DRG neurons project to the superficial laminae of the dorsal horn, particularly laminae I and II [4,5].

DRG neurons process many sensory modalities, such as proprioception and touch, with the exception of small DRG neurons, which primarily conduct pain signals. Thus, DRG neurons can be characterised into four dorsal root fibre types. Aα fibres conduct information from muscle and skeletal mechanoreceptors. Aβ fibres relay signals from cutaneous and subcutaneous mechanoreceptors. Aδ and C fibres conduct nociceptive information [6]. The central terminals from Aδ and C fibres innervate lamina I (marginal zone) and lamina II (substantia gelatinosa). Superficial (lamina I) and deep (lamina V) second order pain transmission neurons in the dorsal horn send projections to brainstem/midbrain nuclei and thalamic nuclei, respectively, then on to sensory and emotional cortical regions [4]. In addition, descending modulatory pathways from brain strongly modulate ascending information. Direct application of conotoxins to the spinal cord is a potential target for Na_V_ channel inhibitors, where specific inhibition of neurotransmission in the central terminals of nociceptive primary afferent nerves may be achieved [7].

Within DRG neurons, there is a multitude of ion channels, receptors and peptides [8,9,10]. These channels and receptors are believed to serve many functions including transduction, conduction and modulation of synaptic transmission [8]. Transduction involves transient receptor potential channels, sodium channels, acid-sensing ion channels, and ATP-sensitive receptors that convert stimuli to electrical signals in the peripheral terminals of DRG neurons. Conduction involves potassium and sodium channels that help propagate action potentials. Synaptic transmission comprises of neurotransmitter release aided by voltage-gated calcium channels (Ca_V_) and glutamate receptors located on presynaptic terminals of primary afferent fibres in the dorsal horn [8]. Among the diverse ion channels differentially expressed in sensory neurons, some Na_V_ channel subtypes are expressed selectively in pain sensing neurons.

## 2. Na_V_ Channels Structure

Na_V_ channels are large complex structures made up of a primary alpha (α) subunit along with one or two auxiliary beta (β) subunits. The primary α subunit is about 260 kDa in size with four homologous domains each with six transmembrane segments (S1–S6), [11,12]. Much of the primary structure of the channel was revealed by crystallisation of the bacterial sodium channel, Na_V_Ab [13]. Na_V_Ab has a central pore surrounded by four pore forming modules made of S5 and S6 segments and a pore loop. The outer edge of the pore is associated with S1–S4 voltage sensing modules. There is also a wide outer vestibule, a narrow ion selectivity filter, a large central cavity and an intracellular activation gate made by crossing of S6 segments [11].

In contrast to the single α subunit, there can be one or two β subunits based on the location of the channel. The brain Na_V_ channels are generally made of two β subunits [11]. The β subunit comprises of a *N*-terminal extracellular immunoglobulin-like fold, a single transmembrane segment and a short intracellular segment. The primary component of the β subunit helps regulate channel kinetics such as voltage dependence of activation and inactivation. The extracellular immunoglobulin domains of β subunits function as cell adhesion molecules that interact with extracellular matrix proteins and other cell adhesion molecules [11].

## 3. Na_V_ Channel Subtypes

There are nine different mammalian sodium channel α-subunits labelled, Na_V_1.1 to 1.9. Despite different isoforms of Na_V_, they share at least 50 percent of their amino acid sequence [14]. When phylogenetically grouped, Na_V_1.1, 1.2, 1.3 and 1.7 show great similarity and can be found on human chromosome 2q23-24 [11,15]. In comparison, Na_V_1.5, 1.8 and 1.9 encoded on human chromosome 3p21-24 are also closely related with 64 percent similarity to Na_V_ channels on chromosome 2. Distinguished from both these groups, Na_V_1.4 is primarily expressed in skeletal muscles and encoded by chromosome 17q23-25 while Na_V_1.6 is mainly found at nodes of ranvier and encoded by chromosome 12q13. However, both Na_V_1.4 and Na_V_1.6 show at least 84 percent similarity to Na_V_ channels coded by chromosome 2 [11,14].

## 4. Roles of Sodium Channels in Nociception and Chronic Pain

In terms of neuropathic and inflammatory pain, it is unlikely that all Na_V_ channels play a role. Within the diverse DRG neuronal population, Na_V_ channels are localised in various subgroups based on function. Therefore, the properties of each Na_V_ channel needs to be explored. Na_V_1.1 is primarily found in large diameter A-fibre DRG neurons suggesting a possible role in proprioception [8]. However, a proportion of small non-peptidergic neurons also contain Na_V_1.1. In fact, mutations of the SCN1A gene that codes for Na_V_1.1 have been associated with familial hemiplegic migraine suggesting a likely role in nociception [8,16]. In rodents that undergo spinal nerve ligation to model nerve injury, Na_V_1.1 levels are downregulated. Hence it is unclear as to what role Na_V_1.1 may play in nociception [8,17]. Although Na_V_1.2 is found in many regions of the central nervous system (CNS), its level remains low in adult DRG neurons. Therefore, Na_V_1.2 channels are unlikely to play a major role in nociception [8,17].

Na_V_1.3 is extensively expressed in neonatal DRG neurons. However, only a little of it can be detected in adult naïve DRG neurons [18]. Following peripheral nerve injury, 37 percent of L5 DRG neurons were Na_V_1.3 positive [19]. Furthermore, 40–47 percent of medium to large DRG neurons were Na_V_1.3 positive after SNL [20]. In spite of these findings, the role of Na_V_1.3 in neuropathic pain remains controversial, as neuropathic pain develops in conditional and conventional Na_V_1.3 knockout mice [8,21]. Moreover, ectopic discharges from injured nerves were unaltered in Na_V_1.3 conventional knockout mice [21]. Thus, it is less likely that Na_V_1.3 plays a major role in the induction of abnormal spontaneous activity in injured neurons. Alternatively, it is possible that the loss of Na_V_1.3 is compensated by other Na_V_ channel subtypes. It therefore remains uncertain whether or not conotoxins that target Na_V_1.3 could be developed as pain therapeutics.

Na_V_1.4 is primarily found in skeletal muscles and Na_V_1.5 is mainly located in cardiac muscles [14]. Both these channels also do not show a large presence within DRG neurons. Low or complete absence in DRG neurons coupled with localisation outside the DRG, makes Na_V_1.4 and 1.5 unlikely candidates to play a role in nociception. In terms of analgesic potential, activity of conotoxins at Na_V_1.4 and 1.5 channels may lead to severe adverse effects.

In contrast, Na_V_1.6 co-localised with the neurofilament-200 (NF200) marker, which binds to sensory neurons that gives rise to myelinated axons [20]. Therefore, it can be assumed that Na_V_1.6 is an A-fibre specific channel, which is not surprising because it is associated with nodes of Ranvier. Following SNL, Na_V_1.6 levels are down-regulated [22]. However, infraorbital nerve injury was found to increase Na_V_1.6 levels proximal to the lesion site [23]. Despite this elevation, it remains to be determined whether Na_V_1.6 plays a role in the abnormal spontaneous activity of injured DRG neurons [8].

Na_V_1.7 shows a wide distribution across different DRG neurons but can be predominantly found in small DRG neurons [8,24]. Since most small DRG neurons are linked to unmyelinated axons, Na_V_1.7 was primarily co-localised with NF-200 negative neurons [20]. Due to a slow closed-state inactivation, Na_V_1.7 is unable to respond during high frequency stimulation [25]. However, small depolarizing stimuli close to resting membrane potential have been shown to activate Na_V_1.7 [8,25]. It may be that Na_V_1.7 is coupled to Na_V_1.8 to boost subthreshold stimuli that activates Na_V_1.8 which recovers from inactivation at a rapid rate and produces high frequency action potentials [6]. Following peripheral inflammation, Na_V_1.7 mRNA and protein levels are upregulated [26,27]. Furthermore, Na_V_1.7 knockout mice do not develop hyperalgesia in pain models [28]. In humans, the loss of function mutation of the SCN9A gene that codes for Na_V_1.7 is linked to channelopathy-associated insensitivity to pain (CIP), while SCN9A gain of function mutations cause paroxysmal extreme pain disorder and primary erythromelalgia [8,29,30]. This evidence suggests that Na_V_1.7 is important for the development of acute and inflammatory pain.

The role of Na_V_1.7 in neuropathic pain seems more complex. In a rat diabetic neuropathy model it was found that both Na_V_1.7 protein and current was elevated [8,31]. However, SNL and spared nerve injury decreased Na_V_1.7 protein levels [8,22,32]. Furthermore, there was a decline in Na_V_1.7 protein levels following peripheral axotomy or traumatic central axotomy in the injured DRG neurons of humans [33]. In mice, SNL induced mechanical allodynia despite conditional knockout of Na_V_1.7 [34]. In contrast, painful neuromas of amputees with phantom limb pain have been found to accumulate Na_V_1.7 protein [8,35,36]. Nonetheless, conotoxins or other peptide toxins that selectively block Na_V_1.7 are likely to have therapeutic benefits in at least some pain states. Additionally, as the only recognised deficit in CIP patients is a loss of smell (anosmia), Na_V_1.7 remains a promising analgesic target with a minimal side effect profile [37].

Na_V_1.8 is primarily found in small diameter nociceptive DRG neurons [8,38]. Carrageenan injection into the hind paw of rodents to induce inflammation up-regulated Na_V_1.8 mRNA and protein [26,39]. Moreover, Na_V_1.8 knockout mice displayed impaired thermal and mechanical pain hypersensitivity following carrageenan induced inflammation [40]. Hence, Na_V_1.8 is widely accepted to play role in inflammatory pain. However, peripheral nerve injury decreased Na_V_1.8 mRNA and protein levels in small diameter injured DRG neurons [8,41]. Additionally, SNL at the L5 level up-regulated Na_V_1.8 in uninjured *C*-fibres of sciatic nerves [8,42]. It is believed that TNFα may be responsible for these elevated Na_V_1.8 levels as inhibition of synthesis and knockout of TNFα eliminated the up-regulation of Na_V_1.8 following nerve injury [43]. The possibility that Na_V_1.8 plays a role in the development in allodynia was reinforced by a study which revealed that MuO-conotoxin MrVIB selectively blocked Nav1.8 sensory neuron specific sodium channels and chronic pain behaviour without motor deficits [7]. Another report showed that mechanical allodynia was attenuated in a dose dependent manner with Na_V_1.8 selective blockade in rats with SNL induced neuropathic pain [44]. Additionally, it was found that two Na_V_1.8 mutations isolated in patients with painful neuropathies enhanced the response of DRG neurons to depolarisation [45]. More intriguing evidence for the role of Na_V_1.8 in neuropathic pain immerged in an investigation of saphenous nerve neuromas [46]. Results revealed that after 22 days from induced axonal damage, 19 percent of fibres in wild type mice neuromas had spontaneous activity compared to almost no spontaneous activity in the Na_V_1.8 null mice. Furthermore, 10 days post-surgery, a significantly higher proportion of fibres were mechanosensitive in wild type mice in comparison to Na_V_1.8 null mice. Thus, it was concluded that Na_V_1.8 is critical for spontaneous activity and that it may induce ectopic mechanosensitivity in sensory axons that are damaged [46]. These results collated together suggests that Na_V_1.8 plays a role in painful neuropathies by promoting neuronal hyperexcitability. However, Na_V_1.8 knockout mice develop neuropathic pain suggesting a possible difference in the role of Na_V_1.8 in neuropathic pain between rats and mice [8,47]. Moreover, Na_V_1.8 polymorphisms have been linked to atrial fibrillation. Atrial fibrillation is an electrical disturbance in the heart leading to inefficiencies in cardiac activity [48]. Therefore, a further complication that needs to be considered is the potential for atrial fibrillation due to Na_V_1.8 block. Overall, Na_V_1.8 is a promising target for inflammatory analgesia. However, its usefulness for neuropathic pain relief is less clear due to differences between rats and mice as well as potential side effects linked to atrial fibrillation.

Na_V_1.9 is selectively found in small-diameter DRG neurons [8]. These channels are known to have slow kinetics and currents that are persistent near resting membrane potential [49]. In the complete Freund’s adjuvant (CFA) model to induce inflammation, level of Na_V_1.9 was significantly elevated [50]. Moreover, Na_V_1.9 knockout mice show decreased pain behaviours to induced inflammation [51]. Therefore, Na_V_1.9 may also play an important role in inflammatory pain. However, immunohistochemical analysis after SNL in rats show decreased Na_V_1.9 levels in the injured DRG neurons [41]. Moreover, mechanical and thermal hypersensitivities developed in Na_V_1.9 knockout mice after spared nerve injury to the same extent as wild type mice [52]. However, when two Na_V_1.9 mutations isolated from patients with painful neuropathies were expressed in Na_V_1.9 knockout mouse DRG neurons, the resting membrane potential was depolarised [53]. Thus, the neurons were more prone to hyperexcitability. Clinically, a heterozygous mutation in Na_V_1.9 was found in two unrelated individuals with congenital inability to sense pain [54]. However, a different Na_V_1.9 mutation was isolated in patients with autosomal dominant episodic pain [55]. Therefore, the nature of the Na_V_1.9 mutation appears to lead to contrasting clinical outcomes. Overall these results indicate that Na_V_1.9 may play a role in neuropathic pain.

## 5. Toxin Interaction with Na_V_ Channels

Most pharmacological agents that interact with Na_V_ channels bind to the α subunit [14]. At least eight different binding sites have been isolated for neurotoxins along with a site for local anesthetic binding. There are a range of animal toxins that block Na_V_ channels. These toxins have been identified in venoms from centipedes, spiders and sea dwelling cone snails. Due to the difficulties of isolating native toxins in large quantities, synthetic forms of the native toxins have been usually developed. The specific binding characteristics of these toxins have made them great tools to identify channel structures.

**Figure 1:**
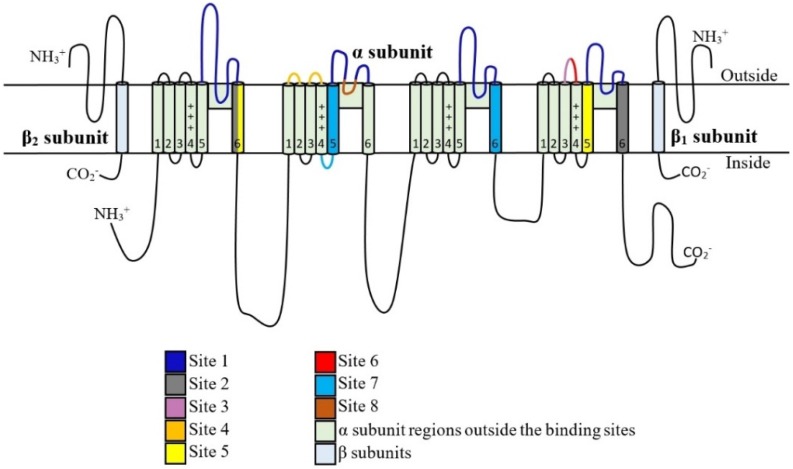
Site 1 binds μ-conotoxins, GIIIA, GIIIB, GIIIC, PIIIA, TIIIA, BuIIIA, BuIIIB, BuIIIC, SmIIIA, SIIIA, KIIIA. Site 2 binds vetridine and shows no major conotoxin interactions. Site 3 interacts with α-scorpion toxins and does not bind conotoxins. Site 4 binds β-scorpion toxins, μO-contoxins, MrVIA, MrVIB and MfVIA as well as ι-conotoxins RXIA, r11b, r11c, r11d and r11e. Site 5 interacts with brevetoxin and ciguatoxin while no conotoxin interactions have been reported. Site 6 binds δ-conotoxins TxVIA, PVIA, SVIE, GmVIA, EVIA, TsVIA and SuVIA. Insecticide binding site 7 does not interact with conotoxins. Site 8 binds the conotoxin, µO§-GVIIJ.

Conotoxins are potent ion channel inhibitors. While there are many different types of conotoxins, four families that target Na_V_ channels with a potential fifth family have been discovered recently. These are identified as µ, μO, δ, ι and µO§. µ and µO-Conotoxins inhibit Na_V_ channels through targeting the channel pore and the voltage sensor, respectively. δ-Conotoxins cause over excitation by slowing inactivation. ι-Conotoxins induce excitotoxicity through shifting the voltage dependence of activation to more hyperpolarised voltages. µO§-Conotoxins cause Na_V_ channel inhibition by mechanisms that are yet to be elucidated. As reviewed elsewhere, it is evident that conotoxins have very diverse mechanisms of action correlated with great structural variability [56]. Therefore, each family of conotoxins need to be studied individually. Identified binding sites on Nav channels are summarized in Figure 1 and conotoxin interactions with these sites are discussed in detail below.

### 5.1. μ-Conotoxins that Interact with Binding Site 1

Site 1 binds tetrodotoxin (TTX), saxitoxin (STX) and μ-conotoxins. This site is largely comprised of the P loops between S5 and S6 in each domain that form part of the outer vestibule of the channel. In general, μ-conotoxins bind more superficially to the channel pore than TTX and STX due to their larger size [57]. However, all three toxins inhibit Na_V_ channel current as these P loops play a critical role in controlling permeation and selectivity [58]. Whilst tetrodotoxin and saxitoxin bind to site 1 on most Nav channel types, µ-conotoxins exhibit selectivity for more restricted subsets of channels as summarized in Table 1.

**Table 1:** μ-conotoxin activity at Na_V_ channels.

μ-Conotoxin Type	*Conus* Species	Primary Na_V_ Channel Targets
GIIIA	*geographus*	Na_V_1.4 [59,60]
GIIIB	*geographus*	Na_V_1.4 [61]
GIIIC	*geographus*	Na_V_1.4 [3,62]
PIIIA	*purpurascens*	Na_V_1.4, 1.2, 1.7 [63,64]
TIIIA	*tulipa*	Na_V_1.4, 1.2 [65,66]
BuIIIA	*bullatus*	Na_V_1.4 [67]
BuIIIB	*bullatus*	Na_V_1.3, Na_V_1.4 [67,68,69]
BuIIIC	*bullatus*	Na_V_1.4 [67]
SmIIIA	*stercusmuscarum*	Na_V_1.8 [70,71]
SIIIA	*striatus*	Na_V_1.2, 1.3, 1.4 [72,73]
KIIIA	*kinoshitai*	Na_V_1.2, 1.3, 1.4, 1.5, 1.6 [74,75]

μ-conotoxins were first isolated from the venom of the fish-hunting snail *C. geographus*. They are a family of 22-residue peptide amides that inhibit sodium channels [62]. μ-contoxins are only one class of paralytic toxins found in this species of cone snails which also produce ω-conotoxins and α-conotoxins that target Ca_V_ channels and acetylcholine receptors, respectively. The first extensively studied μ-conotoxin was GIIIA. It had three hydroxyproline residues and three disulphide bridges. It was shown to compete with TTX and STX for binding at Na_V_1.4 channels but showed no affinity for Na_V_ neuronal subtypes [76]. Nevertheless, the potency of GIIIA varies between the rat and human Na_V_1.4 subtypes increasing from an IC_50_ value of 50 nM in rats to 1500 nM in humans [59]. Moreover, GIIIA shows no inhibition of Na_V_ current in rat DRG neurons [65].

In 1989, GIIIA was synthetically manufactured [60]. The peptide nature of this toxin allowed it to be modified significantly without the loss of biological activity. The three dimensional structure of this toxin revealed that it had an overall disk shape for the backbone with seven Arg and Lys side chains projecting radially. The essential residues for activity were located on one side of the molecule to associate with the receptor site. These key residues important for activity were at positions 13 (Arg13), 19 (Arg19), 16 (Lys16), and 17 (Hyp17 [hydroxyproline]). From these residues, Arg13 was found to be the most significant. The substitution of Arg with Gln at position 13 led to an absence of toxin binding to eel electroplax membranes in a competitive binding assay with STX up to 1 μM [59,77]. Furthermore, Hyp17 in GIIIA is believed to interact with the same Na_V_ channel site as TTX and STX hydroxyl groups [78]. However, GIIIA has some level of redundancy in its residues that interact with Na_V_1.4 as just the presence of R13 and K16 alone can cause channel block [79]. Hence, GIIIA’s primary purpose is to serve as a histochemical marker for Na_V_1.4 channels.

GIIIB has a similar global fold to GIIIA along with very similar backbone confirmations and Na_V_1.4 channel selective activity [61]. In fact, previously mentioned Arg13, Lys16 and Hyp17 residues of GIIIA are also conserved in GIIIB. However, the sequence of GIIIB differs from GIIIA at four positions leading to one more positive charge on GIIIB. These differences include a change to Arg at position 8 and 14 from Lys and Gln respectively, as well as a substitution of Met for Gln at position 18 and Lys for Arg at position 19 [61]. The change at residue 14 has been suggested as the possible reason behind increased toxicity of GIIIB over GIIIA [61]. Nevertheless, not all of these changes in amino acids modify the activity of the toxin. Moreover, when Arg19 was replaced with a Lys on GIIIA, activity was not significantly impaired. This suggested that the conservation of a positive charge was adequate to maintain the original activity [61,77].

μ-conotoxin GIIIC is also another peptide from *Conus geographus* that selectively targets Na_V_1.4 channels [3]. It also has an Arg at position 14 similar to GIIIB but differs from GIIIB at position 18 where it has a Leu instead of Met [62]. However, due to similarity in its activity profile to GIIIA and GIIIB, this toxin does not provide any new insights to Na_V_ channel activity.

μ-conotoxin PIIIA was isolated from *Conus purpurascens*, an Eastern Pacific fish hunting species of cone snails [63]. Similar to GIIIA, it also binds site 1 on Na_V_ channels [63,80]. When rat Na_V_ (rNa_V_) α subunits were expressed in *Xenopus* laevis (*Xenopus*) oocytes, it reversibly inhibited rNa_V_1.4 channels with an IC_50_ value of 41 nM. Unlike GIIIA, it also blocked rNa_V_1.2 channels at an IC_50_ value of 690 nM and rNa_V_1.7 channels at an IC_50_ value of 6.2 μM [64]. Interestingly, PIIIA was more potent at human Na_V_1.7 channel as seen from an IC_50_ value of 3.1 μM [64]. Hence it is possible to utilise the varied potency of PIIIA towards TTX sensitive Na_V_ channels to distinguish different subtypes. This was illustrated in a nerve growth factor (NGF) treated PC12 cell line which had a transient upregulation of Na_V_1.7 in the first 24 h followed by an increase in Na_V_1.2 beyond 24 h. As a result, it was shown that within the first 24 h, only 13% of the observed Na_V_ current was inhibited by PIIIA followed by 39% at 48 h and 63% at 72 h [64]. Thus, PIIIA serves as a valuable probe to distinguish Na_V_ channel subtypes when used in conjunction with other selective toxins.

A µ-conotoxin with a similar single conformation in solution to PIIIA is TIIIA. It was isolated from the venom duct of *Conus tulipa* [65]. At the point of discovery, it had less than 35% sequence homology to other µ-conotoxins. Several residues found in µ-conotoxins that inhibit TTX sensitive Na_V_ channels including Arg14 was conserved in TIIIA. TIIIA potently displaces STX from Na_V_1.2 channels in the rat brain and Na_V_1.4 channels in skeletal muscle. Consistent with low levels of Na_V_1.2 and the absence of Na_V_1.4 channels, 3 µM TIIIA showed no inhibition of Na_V_ current in DRG neurons [65,66]. Therefore, the primary function of TIIIA is to inhibit rat Na_V_1.2 and 1.4 channels outside of DRG neurons.

Another set of μ-conotoxins that target TTX-sensitive Na_V_ currents are BuIIIA, BuIIIB and BuIIIC from the cone snail species *Conus bullatus* [67]. More specifically, BuIIIA inhibited Na_V_1.4 channels expressed in *Xenopus* oocytes by about 87 percent while BuIIIB and BuIIIC inhibited about 96 percent of the current at a concentration of 1 μM. However, BuIIIA block of Na_V_1.4 was reversible with a k_off_ value of approximately 0.021 min^−1^, while BuIIIB and BuIIIC showed little to no recovery in 50 min [67]. Similar to other µ-conotoxins, BuIIIB *C*-terminal residues such as Trp16, Arg18 and His20 are crucial for Na_V_ blockade [68]. However, a unique feature of these toxins that differentiates them from other μ-conotoxins is an *N*-terminal extension forming part of a short α-helix that include Glu3 to Asn8. In fact, mutation of residues two and three in this *N*-terminal extension caused a 40 fold increase in BuIIIB potency from a K_D_ of about 0.2 μM to 0.0048 μM at Na_V_1.3 channels [69]. Furthermore, a negative charge mutation at the N-terminus of BuIIIB with a γ-carboxyglutamate decreased potency while a positively charged 2,4-diaminobutyric acid substitution increased potency towards Na_V_1.3 channels [68]. Therefore, it is evident that the *N*-terminus region is critical to determine toxin potency. In terms of analgesic potential, these BuIIIA, B and C conotoxins do not show great promise due to their activity at Na_V_1.4 channels that may cause many side effects. As discussed above, the potent Na_V_1.3 block by mutated BuIIIB need to be further explored in pain research. But this may not produce analgesia since Na_V_1.3 knockout mice still developed neuropathic pain [8,21].

A µ-conotoxin that is significantly different from the previously described toxins is SmIIIA. Unlike the other TTX sensitive Na_V_ channel inhibitors, SmIIIA blocks TTX resistant Na_V_ channel currents in amphibian DRG and sympathetic neurons [70]. µ-Conotoxin, SmIIIA was isolated from *Conus stercusmuscarum*. Despite having a different target, SmIIIA shares similar characteristics with other µ-conotoxins, such as the arrangement of cysteine residues in the primary sequence and the conserved Arg13. Nonetheless, structural differences in SmIIIA from other µ-conotoxins include the lack of a Hyp residue and the presence of a Trp15 side chain [70,71]. As TTX resistant Na_V_1.8 and Na_V_1.9 channels play a role in pain pathways, it was of interest to further explore this toxin. Unfortunately, later results revealed that 3 µM SmIIIA did not inhibit human Na_V_1.8 channels [65]. This finding suggested that this toxin was unlikely to have therapeutic potential as it was ineffective at human Na_V_ channels.

Other µ-conotoxins that inhibit TTX resistant Na_V_ channels include SIIIA and KIIIA from the fish-hunting cone snails *Conus striatus* and *Conus Kinoshitai*, respectively. Both toxins block TTX resistant Na_V_ currents in neurons of frog sympathetic and dorsal root ganglia (but not in mammals; see below). However, these peptides show poor inhibition of action potentials in frog skeletal muscles. The primary reason for this weak inhibition is most likely due to these action potentials being mediated by TTX sensitive Na_V_ channel currents [72].

Similar to the conotoxin SmIIIA, μ-SIIIA does not have a Hyp residue and adopts a trans conformation [81]. SIIIA shows about 20% inhibition at 5 µM and about 60% percent inhibition at 25 µM of TTX sensitive Na_V_ current in mouse DRG neurons [73]. Furthermore, in a formalin mediated inflammatory mouse pain model, intraperitoneally administered SIIIA showed analgesia at a dose of 10 nmol per animal [73]. However, SIIIA irreversibly blocked Na_V_1.2, 1.3 and 1.4 channels expressed in *Xenopus* oocytes [82]. Additionally, no inhibition was observed at Na_V_1.5, 1.7 or 1.8 channels. This irreversible activity and non-selective profile, limits the analgesic potential of µ-SIIIA. But activity at multiple TTX sensitive channels may be utilized to treat some forms of pain. For example, in an open label trial, intramuscular TTX provided analgesia in 17 out of 31 treatments in 24 patients with cancer related somatic, visceral or neuropathic pain. The achieved analgesia also lasted beyond two or more weeks [83]. In a subsequent double blind, randomised trial, subcutaneous TTX caused analgesia in some patients with cancer pain that did not respond to opioids or other analgesics [84]. Moreover, repeated subcutaneous TTX treatment in a multicentre, open labelled trial proved analgesic and relatively safe in some patients with unrelieved, cancer related pain up to 400 days [85]. Therefore, conotoxins such as µ-SIIIA that inhibit multiple TTX sensitive Na_V_ channels may provide analgesia to some patients. However, the ability of µ-SIIIA to provide pain relief needs to be evaluated alongside the above mentioned, irreversible pharmacological profile at certain Na_V_ channel subtypes.

µ-KIIIA is a 16 residue peptide with three disulphide bridges. It inhibits Na_V_1.2 channels in *Xenopus* oocytes [74]. Previously identified residues important in Na_V_ interactions including Lys7, Trp8, Arg10, Asp11, His12 and Arg 14 were all conserved on KIIIA [86]. In mouse DRG neurons, KIIIA inhibited 80% of the TTX sensitive and 20% of TTX resistant current [75]. When KIIIA was tested on mammalian Na_V_ isoforms expressed in *Xenopus* oocytes, it inhibited Na_V_1.2 channels irreversibly and Na_V_1.6 with partial reversal. Moreover, Na_V_1.5, 1.3 and 1.4 channels in oocytes were also inhibited by KIIIA with progressively increasing potency [75]. Despite this extensive inhibition on multiple Na_V_ channels, KIIIA also showed analgesia in a mouse model of formalin-induced pain without motor impairment. Results revealed that systemic administration of the toxin, decreased the paw-licking frequency of these mice without impairment in motor performance on a rotarod test up to a concentration of 10 nmol [75]. Overall, it appears that both SIIIA and KIIIA have a complex pharmacological profile with possible inflammatory analgesic potential. However, interaction with Na_V_1.5 may preclude further development due to a high likelihood of cardiac side effects.

An interesting aside to µ-KIIIA activity is its ability to bind site 1 and act alongside TTX. When high concentrations of KIIIA was applied to Na_V_1.2 channels expressed in *Xenopus* oocytes there was a residual current that can be abolished by TTX application [87]. However, TTX inhibited the residual current after KIIIA application at a much slower rate than its usual Na_V_ inhibition rate. Additionally, co-application of TTX alongside KIIIA in any order (KIIIA first or TTX first) accelerated the peptide dissociation rate following washout [87]. Therefore, it has been suggested that TTX could move past the bound conotoxin and attach to a deeper site in the outer vestibule of the channel. Hence it is believed that TTX may form a complex with KIIIA bound to Na_V_ channels [88].

### 5.2. Binding Site 2 and 3 with No Major Conotoxin Interactions

Site 2 binds lipid soluble toxins such as vertridine which amplifies channel activation. This site is primarily located around the S6 segments of domain 1 and 4 [89,90]. More specifically vertidine has been found to bind the S6 segment of domain 1 [89,91]. Site 3 binds α-scorpion toxins and sea anemone toxins, which results in slowed coupling of channel activation and inactivation [14]. This site is partially formed by the domain 4 loop that connects S3 to S4 [90,92].

### 5.3. µO- and Possibly ι-Conotoxin Binding Site 4

Receptor site 4 binds β-scorpion toxins that also enhance channel activation similar to site 2 [14]. This site consists of the S3–S4 loop towards the extremities of the S4 segment and the linker between S1-S3 in domain 2 [90,93]. Additionally, due to β toxin Tz1 from Venezuelan scorpion *tityus zulianus* binding, the *C* terminal loop of domain III has also been included as a β-scorpion toxin interaction site [94]. Outside of β-scorpion toxins, site 4 at least partially binds μO-contoxins. Notably, the structure of the pore loop in domain 3 has been identified as an integral component for μO-contoxin binding [95]. Due to the resemblance of cysteine frameworks between µO, δ and ω conotoxins, µO conotoxins could be regarded as an intermediate between δ and ω conotoxins [96].

The most extensively studied µO-contoxins are MrVIA and MrVIB, isolated from the snail hunting species *Conus marmoreus* [97]. The µ component of their name stems from their similarity to the biological activity of µ-conotoxins. The relation to the “O” super family is due to their cysteine knot residue structure [98]. It was found that MrVIA functionally competed with β-scorpion toxin Ts1 while showing no competition with site 1 binding µ-conotoxin, GIIIA [99]. This result was consistent with µO-contoxins having no interactions at site 1 and at least some binding at site 4. But β-scorpion toxins trap the S4 of domain II in an outward position, while µO-conotoxins bind the S4 voltage sensor in an inward position preventing it from swinging open upon depolarisation [99,100]. Further evidence for µO-conotoxin interaction with the voltage sensor emerged in experiments which showed that a depolarisation to +40 mV for 300 ms reversed the Na_V_ inhibition mediated by MrVIA in mammalian cells [99]. In contrast, MrVIB mediated inhibition of Na_V_ current in *Xenopus* oocytes was reversed back to baseline after repeated pulses to +120 mV during washout [101]. Despite the requirement for repeated depolarising steps, these results also suggested that µO-conotoxins associate with the voltage sensor.

µO-conotoxins might also inhibit mammalian Ca_V_ channels at high concentrations. In caudodorsal neurons of freshwater snail *Lymnaea stagnalis* both MrVIA and MrVIB inhibited Na_V_ channels at an IC_50_ of 0.1–0.2 µM while they also blocked fast inactivating Ca_V_ currents above concentrations of 1 µM [96]. Furthermore, the Na_V_ channel current inhibition required extensive washing to restore baseline levels while Ca_V_ inhibition was rapidly reversed [96]. A difference in activity between the two µO-conotoxins discussed here arose when the inhibition of the sustained Ca_V_ current in the snail neurons was investigated. Results showed that MrVIA inhibited some of the sustained Ca_V_ current while MrVIB displayed no inhibition. In mice, MrVIA CNS injection led to extreme reactions such as ataxia and coma at a very low dose of 0.1 nmol. However, intraperitoneal injection showed no distinct change even at doses 100 times greater [97].

An intriguing aspect of MrVIA is its ability to inhibit TTX resistant currents at an IC_50_ value of 82.8 nM in rat DRG neurons [102]. As this TTX resistant component included Na_V_1.8 and 1.9 channels that play an important role in pain pathways, this finding was of great interest. However, it is important to note that µO-conotoxins also inhibited TTX sensitive currents in rat DRG neurons at an IC_50_ value of 1.02 µM [102]. When TTX sensitive, rat Na_V_1.4 channels were expressed in HEK 293 cells, MrVIA and MrVIB inhibited the Na_V_ current at an IC_50_ value of around 265 nm and 222 nm, respectively. Thus both toxins block Na_V_1.4 channels to a similar extent. In comparison, TTX sensitive Na_V_1.2 channels expressed in HEK 293 cells only showed very weak levels of inhibition. Hence it is evident that µO-conotoxins distinguish between Na_V_1.2 and Na_V_1.4 channels.

Another distinction in µO-conotoxins activity arises when different Na_V_ β subunits are expressed alongside the α subunit. In fact, the dissociation constant of MrVIB decreased from 44 nm for the Na_V_1.8 α subunit alone to 1 nM when the β_2_ subunit was co-expressed in *Xenopus* oocytes. This dissociation constant was also different depending on the co-expressed β subunit type where β_1_, β_3_ and β_4_ had dissociation constants of 8.5 nm, 6.5 nm and 3.5 nm, respectively [101]. In contrast, it was seen that the effect of STX at Na_V_1.8 α subunits expressed in *Xenopus* oocytes was minimally altered by the co-expressed β subunit type. Therefore, two alternative explanations are that MrVIB interacts with some component of the β subunit or that the β subunit type allosterically modulates the MrVIB binding site on the α subunit to different extents [101]. This increased effect of MrVIB in the presence of β_2_ subunits is particularly interesting as the β_2_ subunit is upregulated in rat neuropathic pain models [103]. A further testament to the analgesic potential of MrVIB was its ability to inhibit skin flinch sensitivity in a rat local anaesthetic assay to a much greater extent than lidocaine [104]. Moreover, subcutaneous MrVIB infusion also showed analgesia following foot incision in von Frey testing [104]. As discussed above, MrVIB also decreased allodynia and hyperalgesia in neuropathic and chronic inflammatory pain models without motor side effects at sub micromolar concentrations [7]. Thus, MrVIB shows analgesic potential for further development and study [2].

It is important to note that it was difficult to chemically synthesise a sufficient yield of µO-conotoxins. In fact, the previously implemented two-step oxidative folding strategy only produced a very low yield of MrVIB. Moreover, MrVIB did not fold into the native peptide from its linear, reduced form. Therefore, selenocysteine, an uncommon amino acid was introduced as an isoteric replacement for cysteine. This chemical replacement based on the regioselective formation of a diselenide bond in place of a disulfide bond allowed efficient folding with a greater yield [105]. More importantly, the biological activity of the peptide remained unaltered after the selenocysteine substitution.

Outside of MrVIA and MrVIB, a µO-conotoxin that show some analgesic promise is MfVIA [106]. It is a 32 residue peptide isolated from *Conus magnificus*, synthetically manufactured using a novel regioselective approach described elsewhere [106]. Subsequently, to characterize the electrophysiological profile of the peptide, the currents from Na_V_1.4, 1.5, 1.6 and 1.7 channels expressed in HEK 293 and CHO cell lines were recorded in the presences of MfVIA. These results revealed that this toxin inhibited, Na_V_1.4, 1.5, 1.6 and 1.7 at IC_50_ values of 81 nM, 431 nM, 1.2 μM and 2.3 μM, respectively. Additionally, Na_V_1.2 channels had an IC_50_ values around 5.1 μM MfVIA while Na_V_1.8 channels had an approximate IC_50_ value of 158 nM, when expressed in *Xenopus* oocytes [106]. Therefore, it is evident that MfVIA potently inhibits Na_V_1.4 and 1.8 channels. Since Na_V_1.8 is abundant in DRG neurons and show promise as an analgesic target, MfVIA mediated inhibition may provide pain relief. However, the presence of Na_V_1.4 channels in skeletal muscles make it an undesirable target limiting the analgesic potential of MfVIA.

Besides μO-conotoxins, site 4 is also suggested to bind ι-conotoxins due to functional similarities with β-scorpion toxins [107]. This family of peptides have a new pattern of eight cysteine residues and a four disulphide scaffold. First isolated from *Conus radiatus* venom, five different toxins labelled r11a (ι-RXIA), r11b, r11c, r11d and r11e were identified [108]. With the exception of r11e, the other four peptides isolated from this venom had many similarities in their primary sequence. However, there were also differences in the peptide structure. For example, r11c had 8 amino acids between the last intercysteine loop compared to the 10 amino acids found on ι-RXIA, r11b and r11d. Furthermore, within this 10 amino acid stretch, there were 8 sequence variants [108]. The functional activity of these peptides were characterised in a frog cutaneous, pectoris preparation. Results showed that nerve stimulation led to repetitive firing of action potentials in the nerve and muscle following 1 μM r11b application. Moreover, there were spontaneous action potentials in the nerve after r11b exposure.

These results found in the presence of r11b were replicated for ι-RXIA, r11c and r11e. However, ι-RXIA was the most potent and least reversible toxin from this group. ι-RXIA caused many more spontaneous action potentials and consistent action potential trains in comparison to the other toxins in this group [108]. The increased toxic activity of ι-RXIA is attributed to a D-Phe44 positioned third to last on the toxin. These results were further supported by *in vivo* experiments that demonstrated intracranial injections of ι-RXIA caused seizures in mice [107]. 

Subsequent experiments set out to elucidate the mechanisms behind this excitotoxic profile. It was found that ι-RXIA shifted mouse Na_V_1.6 voltage dependence of activation to more hyperpolarised potentials with minimal effects on inactivation [107,109]. Additionally, when ι-RXIA was applied to rat Na_V_ α subunits expressed along with β_1_ in *Xenopus* oocytes, Na_V_1.7, 1.2 and 1.6 channels were sensitive while Na_V_1.1, 1.3, 1.4 and 1.5 channels were insensitive [107]. To assess ι-RXIA effect on Na_V_1.8, the TTX resistant component of small mouse DRG Na_V_ current was isolated. As this TTX resistant current is primarily composed of Na_V_1.8, any effect would be mediated through it. However, results indicated that Na_V_1.8 was fairly insensitive to ι-RXIA [107].

In terms of the ι-conotoxin binding site, several lines of evidence need to be explored further. Firstly, ι-RXIA has a high affinity for Na_V_1.6, similar to β-scorpion toxin Cn2 [110]. Moreover, when the critical ι-RXIA, D-Phe44 residue was modified to L-Phe44, there was an uncoupling between binding and modulation efficacy. In Na_V_1.2 channels, the G845N mutation of the S3–S4 loop in domain 2, decreased the affinity of β-scorpion toxin Css IV by almost 10 fold. This decrease in affinity was coupled with a loss of toxin modulation on channel activation despite activation remaining intact due to the mutation [111]. Thus, channel modification yielded similar results for Css IV as the toxin mutation did for ι-RXIA. For these reasons, it is possible that ι-conotoxins also interact at Na_V_ binding site 4 similar to β-scorpion toxins [107]. Overall, ι-conotoxins provide a diverse set of tools to study excitability in neurons.

### 5.4. Site 5 Does Not Show Major Conotoxin Interactions

Site 5 binds complex polyether toxins such as brevetoxin and ciguatoxin produced by dinoflagellates. In particular, brevetoxin was found to bind S6 of domain 1 and S5 of domain 4 [90]. However, no interactions of conotoxins with site 5 have been reported to date.

### 5.5. δ-Conotoxin Binding Site 6

Neurotoxin binding site 6 is linked to δ-conotoxins that slow the Na_V_ channel inactivation rate. Although the exact location of binding site 6 is unknown, δ-SVIE conotoxin was found to interact with a conserved residue in the S3-S4 linker of domain 4 [100,112]. In contrast to neurotoxins, local anaesthetics and antiarrhythmic drugs interact with an overlapping receptor site within the inner cavity of the channel pore. Three of the four S6 segments in α subunit domains appear to help form this binding site with the S6 segment in domain four playing a prominent role [14,15]. Selectivity of δ-conotoxins for different Nav channel types is shown in Table 2.

**Table 2:** δ-Conotoxin activity at Na_V_ channels.

δ-Conotoxin Type	*Conus* Species	Primary Na_V_ Channel Targets
TxVIA	*textile*	Undetermined (no activity in mammals) [113,114,115]
PVIA	*purpurascens*	Na_V_1.2, 1.4, 1.7 [64,116]
SVIE	*striatus*	Na_V_1.4 [112]
GmVIA	*gloriamaris*	Na_V_1.2, 1.4 [117,118]
EVIA	*ermineus*	Na_V_1.2, 1.3, 1.6 [119]
TsVIA	*tessulatus*	Na_V_1.6 [120]
SuVIA	*suturatus*	Na_V_1.3, 1.4, 1.6 and 1.7 [121]

Similar to other conotoxins, δ-conotoxins are small peptides that are rich in disulphides. As mentioned earlier they increase the time course of Na_V_ channel inactivation [122]. King Kong peptide (TxVIA) from *Conus textile* was the first δ-conotoxin to be isolated from a snail hunting species of cone snails [113]. The unique name “King Kong” peptide stems from the dominant posture that lobsters take following injection of this toxin. It is made up of 27 amino acids with multiple cysteine residues. In fact, this cysteine structure was very similar to that of ω-conotoxin MVIIA. Yet, there was no indication that the King Kong peptide inhibited Ca_V_ channels [113]. This functional difference may be due to modified channel interactions mediated by the charge difference between these two toxins and the significantly more hydrophobic nature of the King Kong peptide. In terms of it biological activity, TXVIA has a complex profile showing activity against some molluscs (molluscicidal), some insects (insecticidal) but no activity towards mammals [113,114,115]. Despite the lack of activity in mammals, TXVIA binds rat brain membranes. [115]. Therefore, TXVIA played a protective role and acted as an antagonist in rat brains when administered *in vivo* with the *Conus striatus* toxin [115]. However, TXVIA is very unlikely to produce pain relief on its own due to lack of activity in mammals.

Two more intriguing δ-conotoxins are PVIA and SVIE isolated from the fish hunting, *Conus purpurascens* and *Conus striatus*, respectively [116,122]. Both toxins slowed the Na_V_ time course of inactivation. However, they differed in the reversal of inhibition where δ-SVIE mediated activity was mostly irreversible while δ-PVIA effects were reversed with washout. An additional difference between these two conotoxins was the level of toxin activity on inactivation as a result of an 80 mV conditioning depolarisation for 300 ms. These results showed that δ-PVIA activity on Na_V_ inactivation was significantly decreased by the conditioning depolarisation in comparison to δ-SVIE activity which was largely unaffected [122]. In *Xenopus* oocyes, δ-PVIA inhibited rat Na_V_1.2, 1.4 and 1.7 channels at progressively higher concentrations [64].

When δ-SVIE was tested on Na_V_1.4 channels expressed in HEK 293 cells, it was found to be a potent gating modifier [112]. More specifically, rapid channel inactivation was slowed by the toxin yielding a K_D_ value of around 500 nM. The specific interaction of the toxin with Na_V_1.4 channels was believed to be dependent on 3 hydrophobic residues identified as Y1433, F1434 and V1435 on the S3/S4 linker of domain 4. However, the analgesic potential of δ-SVIE is very limited due to its activity at Na_V_1.4 channels.

δ-GmVIA is a conotoxin from the mollusk-hunting snail *Conus gloriamaris* that induces convulsions in land snails [117,118]. In sea hare *Aplysia* abdominal ganglia cells, δ-GmVIA shifted the Na_V_ voltage dependence of steady state inactivation to more depolarised potentials and the steady state activation curve to more hyperpolarised potentials. When the toxin was tested on Na_V_ channels expressed in *Xenopus* oocytes it inhibited Na_V_1.2 and 1.4 channels. Upon structural analysis, a similar cysteine framework to the King Kong peptide and Ca_V_ inhibiting ω-conotoxins was identified. Overall this toxin was very unlikely to have any analgesic potential due to its activity on Na_V_1.4 channels [117].

δ-EVIA from *Conus ermineus* is a 32 amino acid with a six cysteine and four-loop framework similar to omega conotoxins [119]. In amphibian myelinated axons and spinal neurons, δ-EVIA increased the duration of the action potentials by inhibiting Na_V_ channel inactivation. Upon application, δ-EVIA was also found to act on rat Na_V_1.2, 1.3 and 1.6 channels while showing no activity at Na_V_1.4 or Na_V_1.5 channels expressed in *Xenopus* oocytes. Moreover, when injected intracerebroventricularly, low doses (40 pmol) of δ-EVIA produced hyperactivity and seizures within 1–3 min. High doses (1 nmol) of δ-EVIA were found to be lethal [119]. These results show potential for δ-EVIA to be used as a tool for differentiating Na_V_ channel subtypes.

Recently isolated δ-TsVIA conotoxin was sourced from the venom of *Conus tessulatus* [120]. It is a 27 amino acid peptide that inhibits TTX sensitive Na_V_ current. Relative to TTX, δ-TsVIA demonstrated slow dissociation kinetics in calcium imaging experiments of mouse DRG neurons. Furthermore, when mouse Na_V_1.6 α subunits were co-expressed with rat Na_V_β1 subunits in *Xenopus* oocytes, the rapid channel inactivation was inhibited by δ-TsVIA [120]. Future studies to evaluate the analgesic potential of this venom peptide, need to determine the Na_V_ channel subtype specificity and reversibility of inhibition.

δ-Conotoxin SuVIA was isolated from the worm hunting *Conus suturatus* [121]. It was found to activate human Na_V_1.3, 1.4, 1.6 and 1.7 channels expressed in HEK 293 cells. Further electrophysiological analysis revealed that δ-SuVIA increased peak Na_V_1.7 channel current by about 75 percent and shifted the voltage dependence of activation to −15 mV from −25 mV. Half maximal activation voltage was also shifted in a hyperpolarising direction by about 9.5 mV [121]. These changes in activation coupled with a depolarising increase of about 2.8 mV in half maximal inactivation voltage would underlie the toxin mediated enhancement of Na_V_1.7 current. Overall, δ- SuVIA does not show any analgesic potential due to the absence of inhibitory activity.

### 5.6. Site 7 Does Not Show Major Conotoxin Interactions

This site is positioned in a hydrophobic cavity surrounded by the domain II S4–S5 linker and S5-helix as well as domain III, S6 helix. Site 7 is associated with binding insecticides such as pyrethroids and DDT [123]. However, there are no identified conotoxin interactions with this binding site.

### 5.7. µO§-GVIIJ Conotoxin Binding Site 8

Binding site 8 is located around the pore loop of domain II [124]. This site was identified from analogues of µO§-GVIIJ conotoxin that required Cys910 residue near the Na_V_ pore loop to be disulphide tethered to Cys24 on the toxin. As µO§-GVIIJ conotoxin causes Na_V_ channel inhibition, it should be noted that site 8 differs from the binding sites for µ-conotoxins and µO-conotoxin MrVIB which also cause Na_V_ channel inhibition. To functionally verify different binding sites, it was shown that the residual current following µ-conotoxin KIIIA application on to Na_V_1.2 channels was blocked by 3 µM GVIIJ_SSG_ (µO§-GVIIJ conotoxin analogue). In the simultaneous presence of GVIIJ_SSG_ and µO-MrVIB, depolarising pulses caused µO-MrVIB to dissociate from the Na_V_1.2 at an accelerated rate. The remaining component following depolarisation was identical to the residual current that was detected after the application of GVIIJ_SSG_ alone. Therefore, it was evident that GVIIJ_SSG_ was bound at a separate site to µO-MrVIB [124].

µO§-GVIIJ is a conotoxin extracted from *Conus geographus* [124]. Activity of this conotoxin is determined by two derivatives known as GVIIJ_SSG_ and GVIIJ_SH_ due to the limited availability of the native peptide. A primary difference between these two analogues is based around Cys24 which is in a disulphide linkage on GVIIJ_SSG_ in comparison to a free thiol form on GVIIJ_SH_. Therefore, GVIIJ_SH_ served as a reference to discover the role of the disulphide linkage on Cys24. GVIIJ_SSG_ inhibited all TTX sensitive rat Na_V_ channel subtypes expressed in *Xenopus* oocytes at K_D_ values that ranged from 5 to 360 nm. More interestingly, the IC_50_ value at Na_V_1.5 channels was over 200 µM while no Na_V_1.8 block was detected even at concentrations of 100 µM [124]. Upon analysis of toxin dissociation in oocytes expressing Na_V_1.2, GVIIJ_SSG_ block reversed slowly with washout while GVIIJ_SH_ had a two phase recovery which included a rapid phase followed by a slower one. In terms of recovery from inhibition, the native µO§-GVIIJ had a similar slow reversal of inhibition as seen in the presence of GVIIJ_SSG_.

When human Na_V_ channel subtypes were expressed in HEK 293 and CHO cell lines, both GVIIJ_SSG_ and GVIIJ_SH_ caused inhibition of Na_V_ 1.2, 1.4, 1.6 and 1.7 channels at nm ranges. At a concentration of 10 µM, GVIIJ_SSG_ inhibited Na_V_1.5 by 19% while GVIIJ_SH_ only showed 0.3% inhibition. In contrast, Na_V_1.1 and 1.3 channels were only inhibited by GVIIJ_SH_. Overall, GVIIJ_SH_ was more potent at all the channels tested in comparison to GVIIJ_SSG_ [124].

Subsequent experiments set out to identify the effect of β subunits on GVIIJ_SSG_ activity [124]. These results revealed that when rat Na_V_1.7 α subunit was expressed with rat β_1_ or β_3_ subunit, it was inhibited by GVIIJ_SSG_ while the presence of β_2_ or β_4_ subunit, led to toxin resistance. Additionally, chimeras of β_1_ and β_2_ subunits were made by joining the extracellular and transmembrane domains of one β subtype with the intracellular domain of the other β subtype. When co-expressed with Na_V_1.7, these chimeras provided the means to identify the β_2_ region that induced resistance. Thus, results showed that the extracellular domain of β_2_ was critical to induce toxin resistance [124]. The authors suggested that the primary difference between the β_2_ and β_4_ subunits that induce resistance compared to β_1_ and β_3_, is the presence of a disulphide bond with Na_V_1 in the resistant subunits. Therefore, the Cys910 identified earlier may be disulphide linked to its β subunits. Overall, µO§-GVIIJ conotoxin provides another pharmacological tool to study Na_V_ activity.

### 5.8. Local Anesthetic Binding Site

The final Na_V_ binding site is designated for local anesthetics. It is located in the α helix of domain IV segment 6 [125]. Local anaesthetics such as lignocaine do not show great Na_V_ subtype selectivity and yet produce analgesia without a severe side effect profile [126]. In automated patch clamp experiments that measure use dependent inhibition, lignocaine showed no selectivity between Na_V_1.1 to 1.7 channels [127]. Despite the lack of selectivity, multiple studies have reported the use of lignocaine for pain relief. In a randomized, double blind, placebo controlled trial of neuropathic pain sufferers due to peripheral nerve injury, intravenous lignocaine showed significant decreases in pain scores compared to placebo [128]. Side effects, such as lightheadedness, were observed in about half of the 11 tested patients. One patient suffered from nausea. Outside of pain relief, there was also a significant reduction in allodynia. In fibromyalgia, where patients suffer from widespread chronic pain and tenderness, three out of eleven tested women reported 50% pain relief that lasted 4–7 days following local anesthetic application [129].

Multiple factors are attributed to the absence of major side effects following lignocaine treatment. For example, channel block is enhanced at more depolarized voltages. This suggests that the local anesthetic would only be primarily active during pain transmission. Moreover, channel block accrues following a train of brief depolarizing pulses. Therefore, repeated firing neurons would be inhibited to a greater extent in comparison to regular firing neurons. Additionally, channel block is dependent on the frequency of stimulation [127]. Overall, these properties of local anesthetic mediated inhibition hinge on state dependence where the channel is preferentially inhibited in its inactivated state [127]. Therefore, local anesthetics can be utilized as analgesics for certain pain conditions at a tightly regulated dose. Although local anesthetics show promise as analgesics, their activity at Na_V_1.4 and 1.5 channels create significant risks.

## 6. Future of Conotoxins As Potential Analgesics

Outside of Na_V_ channels, conotoxins bind voltage gated calcium (Ca_V_) channels, voltage gated potassium (K_V_) channels and many other targets [3]. Therefore, it is important to highlight that the analgesic targets for conotoxins spread beyond Na_V_ channels. In fact, the only conotoxin approved for clinical use is ω-conotoxin MVIIA (Prialt) which mediates its activity through Ca_V_2.2 channels [130]. Unfortunately, Prialt had many side effects. These effects ranged from whole body shakes driven by possible cerebellar motor defects in rats to neurological impairments that included hallucinations in humans [131]. Since Prialt cannot cross the blood brain barrier, it needs to be administered intrathecally [131]. As a result of complex side effects and a difficult route of administration, Prialt has remained a last resort analgesic for chronic pain sufferers. Hence future conotoxin derived analgesics would need to address the shortcomings of Prialt. Because Na_V_ channels are involved in nociceptive neurotransmission throughout sensory nerves, conotoxins appropriately targeting subtypes of these channels should have the advantage of activity via systemic administration or targeted administration to peripheral tissues.

As discussed here, there is evidence to suggest that Na_V_ channels are intimately involved in all different forms of pain. From the Na_V_ targeting conotoxins, it is evident that μ, μO and µO§-conotoxins are more likely to produce analgesia through inhibition of Na_V_ channels involved in nociceptive transduction and transmission. However, within each conotoxin family there is a great number of Na_V_ targets that will need to be refined through structural modification before therapeutically useful agents can be developed. In general, combinations of Na_V_1.1, 1.3, 1.7, 1.8 and 1.9 channels should be targeted for pain treatments. It may eventuate that the optimal analgesic Na_V_ inhibitors target multiple Na_V_ subtypes among this group. However, different Na_V_ channels play a more substantial role based on the location and type of pain as highlighted in section four. So pain type specific agents may be needed. Perhaps one pitfall of conotoxins that are significantly more selective for one Na_V_ channel subtype is the possibility of redundancy within the system that allows other Na_V_ channel subtypes to compensate for the loss of activity. The studies on pain relief mediated by TTX and local anesthetics such as lignocaine which affect a broad range of Na_V_ channels suggest that inhibiting multiple Na_V_ channels may be an effective approach. In fact, non-selective Na_V_ channel inhibitors such as mexiletine have been shown to be effective in the treatment of neuropathic pain due to peripheral nerve damage and diabetic neuropathies [126,132,133]. However, compounds that target multiple Na_V_ channels have a significant risk associated with them, as they affect skeletal muscle related Na_V_1.4, cardiac contraction associated Na_V_1.5 and nerve conduction linked Na_V_1.6 channels. Therefore, a potential analgesic conotoxin should avoid Na_V_1.4, 1.5 and possibly Na_V_1.6 channels to not to minimize the possibility of adverse side effects. In general, future studies should explore conotoxins that target combinations of Na_V_ channels to attain effective pain relief with a minimal side effect profile.
